# A genome wide association study for backfat thickness in Italian Large White pigs highlights new regions affecting fat deposition including neuronal genes

**DOI:** 10.1186/1471-2164-13-583

**Published:** 2012-11-15

**Authors:** Luca Fontanesi, Giuseppina Schiavo, Giuliano Galimberti, Daniela Giovanna Calò, Emilio Scotti, Pier Luigi Martelli, Luca Buttazzoni, Rita Casadio, Vincenzo Russo

**Affiliations:** 1 Department of Agricultural and Food Science and Technology, University of Bologna, Viale Fanin 46, Bologna, 40127, Italy; 2 Centre for Genome Biology, University of Bologna, Bologna, 40126, Italy; 3 Department of Statistical Sciences “Paolo Fortunati”, University of Bologna, Via delle Belle Arti 41, Bologna, 40126, Italy; 4 Biocomputing Group, Department of Biology, Geology and Environmental Science, University of Bologna, Via San Giacomo, Bologna, 40126, Italy; 5 Consiglio per la Ricerca e la Sperimentazine in Agricoltura, Centro di Ricerca per la Produzione delle Carni e il Miglioramento Genetico (CRA-PCM), Via Salaria 31, Monterotondo Scalo, Roma, 00015, Italy

**Keywords:** GWA, Backfat, Fatness, Obesity, Heavy pig

## Abstract

**Background:**

Carcass fatness is an important trait in most pig breeding programs. Following market requests, breeding plans for fresh pork consumption are usually designed to reduce carcass fat content and increase lean meat deposition. However, the Italian pig industry is mainly devoted to the production of Protected Designation of Origin dry cured hams: pigs are slaughtered at around 160 kg of live weight and the breeding goal aims at maintaining fat coverage, measured as backfat thickness to avoid excessive desiccation of the hams. This objective has shaped the genetic pool of Italian heavy pig breeds for a few decades. In this study we applied a selective genotyping approach within a population of ~ 12,000 performance tested Italian Large White pigs. Within this population, we selectively genotyped 304 pigs with extreme and divergent backfat thickness estimated breeding value by the Illumina PorcineSNP60 BeadChip and performed a genome wide association study to identify loci associated to this trait.

**Results:**

We identified 4 single nucleotide polymorphisms with P≤5.0E-07 and additional 119 ones with 5.0E-07<P≤5.0E-05. These markers were located throughout all chromosomes. The largest numbers were found on porcine chromosomes 6 and 9 (n=15), 4 (n=13), and 7 (n=12) while the most significant marker was located on chromosome 18. Twenty-two single nucleotide polymorphisms were in intronic regions of genes already recognized by the Pre-Ensembl Sscrofa10.2 assembly. Gene Ontology analysis indicated an enrichment of Gene Ontology terms associated with nervous system development and regulation in concordance with results of large genome wide association studies for human obesity.

**Conclusions:**

Further investigations are needed to evaluate the effects of the identified single nucleotide polymorphisms associated with backfat thickness on other traits as a pre-requisite for practical applications in breeding programs. Reported results could improve our understanding of the biology of fat metabolism and deposition that could also be relevant for other mammalian species including humans, confirming the role of neuronal genes on obesity.

## Background

Fat deposition is a key biological process that has important similarities between humans and pigs, potentially useful to elucidate mechanisms determining human obesity. This trait has practical and economical implications in pig breeding as it indirectly affects feeding efficiency and determines carcass value and consumers’ acceptance of pork.

Following consumer demands, breeding goals for fresh pork generally aim at reducing carcass fatness and increasing lean meat content which has adversely affected pork quality (e.g. [1]).

The Italian pig breeding industry is mainly devoted to the production of high quality Protected Designation of Origin (PDO) dry cured hams for which pigs are raised until they reach about 160 kg live weight and appropriate fat coverage of the hams is required [2,3]. Therefore, breeding objectives aim at maintaining fat coverage measured as backfat thickness (BFT). This objective has shaped the genetic pool of Italian heavy pig breeds for a few decades.

To investigate molecular genetic aspects of fat deposition in these pigs, we have recently applied a systematic candidate gene approach and have identified tens of single nucleotide polymorphisms (SNPs) associated with BFT and/or intermuscular fat content in Italian Large White and Italian Duroc pigs [4-10]. For example, a list of more than 30 SNPs has been associated with BFT in Italian Large White, including SNPs already found by other authors in *IGF2*[11], *MC4R*[12], *TBC1D1*[8], *PPARG*[13] genes or newly identified in the *PCSK1*[14], *ACP2*, *CALR*, *JAK3*, and *NT5E*, among several other genes [10]. Moreover, many other SNPs in additional candidate genes have been shown to explain a proportion of genetic variability of fat deposition traits in pigs [4,15,16]. In addition, a large number of QTLs for a variety of fat deposition and related traits have been already reported and listed in the Pig QTL database [17,18].

Recently, with the development of a commercial high throughput SNP genotyping tool in pig (PorcineSNP60 BeadChip [19]), a number of genome wide association (GWA) studies have been carried out in this species focusing on reproduction [20,21], boar taint [22,23], disease resistance [24], structural and body composition, including BFT [25].

We have herein applied a selective genotyping approach in the Italian Large White pig breed and genotyped extreme and divergent pigs for BFT estimated breeding value (EBV) by the Illumina PorcineSNP60 BeadChip ( http://www.illumina.com) tool to identify chromosome regions and markers associated with BFT.

## Results and discussion

### SNP data

A total of 304 performance tested Italian Large White pigs were genotyped with the Illumina PorcineSNP60 BeadChip, interrogating 62,163 loci. One pig was excluded from further analysis due to a call rate below 90%. A call rate ≥0.90 was obtained for 58,680 SNPs (for 2,293 SNP, call rate was 0.0; 1,190 SNP had 0.0<call rate<0.90). About 15.8% (9,287 SNPs) of these potentially useful SNPs had a minor allele frequency <0.05 and were discarded. The remaining 49,393 SNPs were re-mapped on the Sscrofa10.2 genome assembly.

### Genome wide association (GWA) results

Only individuals with extreme phenotypes were genotyped for association study. Several authors have shown that this approach allows to attain the same power with less genotyped individuals (e.g. [26-28]). A recent GWA study for human obesity showed that this design can obtain very similar results to previous studies on general body mass index performed on unselected cohorts of tens of thousands of subjects [29].

In our study, genotyped pigs had extreme and divergent EBV for BFT: 151 had the lowest (thinnest BFT) and 152 the highest (thickest BFT) EBV. These animals were two generation unrelated gilts taken from the performance test of the National selection program of the Italian Large White breed carried out by the National Pig Breeders Association (ANAS).

**Figure 1:**
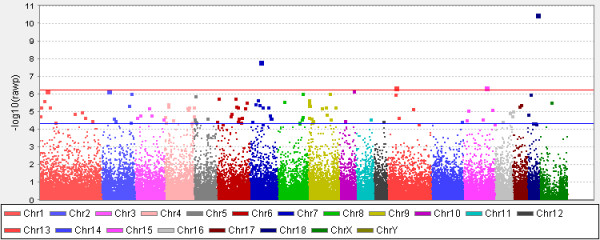
**Manhattan plot of genome wide association results for backfat thickness in Italian Large White pigs.** Red line: significance threshold (P=5.0E-07). Blue line (P=5.0E-05): threshold for suggestively significant results (5.0E-07<P<5.0E-05).

Figure 1 reports a Manhattan plot showing significant (P≤5.0E-07) and suggestively significant (5.0E-07<P≤5.0E-05) SNPs (P_nominal value_ thresholds for significant results were those indicated by the Wellcome Trust Case Control Consortium, WTCCC [30]). Using these values, 4 SNPs were significantly associated (Table 1) whereas 119 SNPs were suggestively associated with BFT (Additional file 1: Table S1).

**Table 1:** Significant SNPs (P<5.0E-07), their chromosome positions and their closest genes in Sscrofa10.2 (Pre-Ensembl)

**SNP**	**CHR:position**^**1**^**(Sscrofa10.2)**	**CHR:position**^**1**^**(Sscrofa10)**	**CHR:position**^**1**^**(Sscrofa9.2)**	**P**^**2**^	**FDR**^**3**^	**SNP position/distance**^**4**^	**Closest gene coordinates**	**Closest gene protein in Pre-Ensembl**	**Gene symbol**
ALGA0098168	18:45408799	18:44540120	18:25516667	3.07E-11	1.51E-06	Intron 3	18:45404849-45567252	ENSSSCP00000017656	*PDE1C*
M1GA0010276	7:50272760	7:50024255	7:50440974	1.45E-8	3.58E-04	3657	7:50276417-50297466	ENSSSCP00000001892	*CRISP1*
ALGA0109557	15:107079255	15:102388547	-	3.81E-7	4.17E-3	4609542	15:102429318-102469713	ENSP00000351255	*STAT4*
ALGA0069549	13:37851945	13:37353675	13:23719928	3.87E-7	4.17E-3	20143	13:37872088-37876725	ENSSSCP00000012196	*STAB1*

^1^Chromosome and nucleotide position in the different genome versions.

^2^P-raw value.

^3^False Discovery Rate.

^4^Localization of the SNP in the corresponding gene or distance from the closest gene (in bp).

The WTCCC criteria to reduce the number of false positive are rather conservative: the suggestive threshold for significance (P=5.0E-05) corresponds to a FDR of 0.02. Had we assumed a false discovery rate (FDR) of 0.05 (P_nominal value_ = 0.000412), a total of 410 SNPs would had been below this threshold and therefore considered at least “suggestively associated” (data not shown).

Single nucleotide polymorphisms with P≤5.0E-05 were located in all porcine autosomal chromosomes (SCC), and on SSCX, and 5 SNPs were in unassembled scaffolds of the Sscrofa10.2 genome version. Among the mapped SNPs, the largest number was on SSC6 and SSC9 (n = 15), SSC4 (n = 13), SSC7 (n = 12) and SSC1 (n = 11) (Additional file 1: Table S1). Twenty-two SNPs were in intronic regions of recognized genes in the Pre-Ensembl Sscrofa10.2 assembly. The closest gene for the remaining mapped SNPs (n = 96) was located in a range from 481 bp to 4.69 Mb (mean = 287.6 kb ± 580 kb, median = 88 kb).

The most significant SNP (ALGA0098168; P=3.07E-11) was on SSC18 (Table 1). This SNP was localized in intron 3 of the phosphodiesterase 1C, calmodulin-dependent 70kDa (*PDE1C*) gene. PDEC1, highly expressed in brain and heart, is involved in the regulation of the cellular level of adenosine 3^′^,5^′^-cyclic monophosphate (cAMP) and guanosine 3^′^,5^′^-cyclic monophosphate (cGMP) that play critical roles in signal transduction [31]. The second most significant SNP (M1GA0010276; P=1.45E-08) was localized on SSC7 at about 3.6 kb from the cysteine-rich secretory protein 1 (*CRISP1*) gene whose known function in reproduction processes is not directly linked to any fat or energy related biological function. The third top SNP (ALGA0109557; P=3.81E-07) was mapped on SSC15 at about 460 kb from the signal transducer and activator of transcription 4 (*STAT4*) gene. STAT4 is a member of the STAT family of transcription factors that transduces interleukin and type 1 interferon cytokine signals in T cells and monocytes, leading to important immunological functions. Reduction of STAT4 activation has been proposed to control obesity-induced inflammation [32]. The fourth most significant marker (ALGA0069549; P=3.87E-07) was located on SSC13 at about 20 kb from the stabilin 1 (*STAB1*) gene. Another close marker (ALGA0109216; at position 38330168 of SSC13; Additional file 1: Table S1) was suggestively significant (P=1.01E-06). The SSC13 region bracketed by these two SNPs includes the *STAB1*-nischarin (*NISCH*) gene interval that in human has been shown to be associated with waist-hip ratio (a measure of body fat distribution) [33].

Several other genes close or within the additional suggestively significant SNPs (Additional file 1: Table S1) have been already involved in obesity related biological mechanisms. Among this list it is worth mentioning: ATP-binding cassette, sub-family B (MDR/TAP), member 1 (*ABCB1*) gene (SSC9; ALGA0109564, P=9.01E-07) whose altered function contributes to steatosis and obesity in mice [34] and a polymorphism in this gene has been associated with obesity risk in Japanese subjects [35]; galanin receptor 3 (*GALR3*) gene (SSC5; M1GA0007458, P=1.25E-06) that is upregulated in adipose tissues of mice fed a high fat diet [36], and whose function is to bind galanin, a neuropeptide that regulates food intake, neurogenesis, memory, and gut secretion; olfactory receptor genes (two genes on SSC9, *OR52N2* and *OR56A3*) have been associated with eating behaviour and adiposity in humans [37]; Parkinson protein 2 (*PARK2*) gene on SSC1 (ALGA0108518, P=5.48E-06) that is regulated in a lipid-dependent manner and modulates systemic fat uptake via ubiquitin ligase-dependent effects [38]; phosphodiesterase 4B, cAMP-specific (*PDE4B*) on SSC6 (ALGA0109354, P=5.95E-06) that has been already shown to be associated with BFT in pigs as well as with obesity in humans [39]; vacuolar protein sorting 13 homolog B (yeast) (*VPS13B*) on SSC4 (ALGA0024658, P=3.00E-05) that causes Cohen syndrome, characterized by truncal obesity [40]; iroquois homeobox 3 (*IRX3*) gene on SSC6 (M1GA0008432, P=4.66E-05), that is involved in the stress response after fat loss [41] and could be linked to obesity and type 2 diabetes through its pancreatic function [42]. Interestingly the second closest gene to this latter SSC6 SNP was *FTO*, that is well known to affect human obesity (i.e. [43]).

Even though the annotation of the pig genome available at present in Pre-Ensembl should be considered preliminary, we further evaluated the potential functional role of regions around associated or suggestively associated SNPs with BFT in our pig population (Additional file 1: Table S1). For this evaluation we used Gene Ontology (GO) information of their corresponding closest genes. Table 2 reports GO terms enriched in this dataset. Interestingly, most of the statistically significant GO terms were related to nervous system development and regulation. This indication might support and extend the role of the nervous system in the genetic predisposition of fat accumulation in mammals, as in part reported in large GWA studies in humans [44] and, subsequently, in pigs [39]. Among the genes listed in these neuronal GO categories (Table 2), few have been already reported to be indirectly associated or involved in obesity related traits. Apart from those already described above (*IRX2* and *PARK2*), it is interesting to mention the delta-like 1 (Drosophila) gene (*DLL1*) as this gene is located in a quite large region (~1 Mb) associated to type 1 diabetes on human chromosome 6 [45]. For several other genes involved in neuronal processes, at present, there is no direct reported link with obesity or fat metabolism. It would be important to further explore their role in affecting the investigated phenotype as a possible strategy to identify new pathways and mechanisms affecting fat deposition. For example, it could be possible to speculatively suggest a relationship between dysbindin (DTNBP1), involved in the modulation of glutamatergic neurotransmission in the brain, schizophrenia and obesity [46]. 

**Table 2:** Results of the Gene Ontology (GO) analysis including closest genes to SNP with P<5.0E-05

**Go sub-ontology**	**GO term accession**	**GO term description**	**Number of involved genes**	**Involved genes**	**DAVID P-value**
Biological Process	GO:0050767	regulation of neurogenesis	5	*ACTR3, LINGO1, IRX3, XRCC2, DLL1*	0.015
Biological Process	GO:0006928	cell motion	8	*ACTR3, CXCR4, SPOCK1, SCNN1G, IL12B, APBB2, ELMO1, CTNNA2*	0.018
Biological Process	GO:0051960	regulation of nervous system development	5	*ACTR3, LINGO1, IRX3, XRCC2, DLL1*	0.024
Biological Process	GO:0060284	regulation of cell development	5	*ACTR3, LINGO1, IRX3, XRCC2, DLL1*	0.029
Biological Process	GO:0030182	neuron differentiation	7	*LINGO1, CXCR4, MTPN, APBB2, OLFM3, NTM, CTNNA2*	0.038
Biological Process	GO:0045664	regulation of neuron differentiation	4	*ACTR3, LINGO1, IRX3, DLL1*	0.040
Biological Process	GO:0048666	neuron development	6	*LINGO1, CXCR4, APBB2, OLFM3, NTM, CTNNA2*	0.044
Molecular Function	GO:0031420	alkali metal ion binding	5	*KCNK9, KCNT2, ATP1B3, SLC22A4, SCNN1G*	0.031
Molecular Function	GO:0000166	nucleotide binding	19	*RBM24, XRCC2, SUCLG2, PKN2, ABCB1, ACTR3, MAP3K5, KCNT2, HIPK1, ASCC3, PDE1C, CELF4, SLC22A4, CELF2, DPYD, RAB38, DOCK10, ARL4C, MOCS1*	0.038
Cellular Component	GO:0043005	neuron projection	7	*NUMA1, CXCR4, MTPN, PARK2, APBB2, DTNBP1, CTNNA2*	0.009
Cellular Component	GO:0031252	cell leading edge	4	*ACTR3, CXCR4, APBB2, CTNNA2*	0.037

### Comparison with other studies in pigs

We compared our GWA results with results obtained in our previous candidate gene studies for BFT in pigs [4-10] and those obtained by other GWA [25] and QTL mapping studies. In our previous studies [5,10], the *IGF2* intron3-g.3072G>A mutation [11] was the most significant marker (P < 1.00E-50 by selective genotyping [10]). As the *IGF2* gene is not assembled in the Sscrofa10.2 genome version, it was not possible to obtain a direct comparison with results obtained for SNPs mapped on SSC2 included in the Illumina PorcineSNP60 BeadChip. However, no SNP in the region where *IGF2* is likely to be found (0–10 Mb) reached the significance level of P<5.0E-05 (Additional file 1: Table S1). Only one SNP (ASGA0008884, position 9139348; P=2.12E-04) was included in the list of markers with P_FDR_<0.05. Several other SSC2 SNPs were suggestively significant (Additional file 1: Table S1) indicating that they might pick up other regions affecting fatness as already reported by QTL studies (e.g. [47,48]) or candidate gene studies [5,10,49,50].

The second most significant marker of our previous candidate gene investigation was the *MC4R* p.Asp298Asn substitution [10]. In the current GWA study, no significant or suggestively significant SNPs were located in the SSC1 region around the *MC4R* gene, even if a few markers had P<1.0E-3 (data not shown). The GWA study by Fan et al. [25], conducted on gilts of a commercial breeding stock, showed that markers around *MC4R* were significantly associated with 10^th^ rib and last rib backfat. These slight differences in terms of level of significance of the markers between the two studies might be due to different *MC4R* haplotype structures in the two pig populations (Fontanesi et al. submitted) or to different positions in the pig body where BFT measurements were taken. However, in general, few results we obtained confirmed those previously obtained by Fan et al. [25] in their GWA study on BFT. This could be due to different experimental designs, incomplete power in the two studies, and/or to differences between the investigated populations. Other results we previously obtained in candidate gene studies (i.e. [10]) could be confirmed if we relaxed the significance threshold up to FDR <0.05 (data not shown).

QTLs for fat deposition traits can be found over all pig chromosomes. Many different studies have repeatedly reported the presence of complex QTL patterns for fat related traits in SSC1, SSC2, SSC4, SSC6 and SSC7 [18]. In the present GWA study, SSC4, SSC6, SSC7, and also SSC9 resulted to be rich in significant or suggestively significant markers (SSC4: expected proportion = 0.068, observed = 0.110; SSC6: expected = 0.059, observed = 0.127; SSC7: expected = 0.063, observed = 0.102; SSC9: expected = 0.061, observed = 0.127). These results seem to indicate these chromosomes to support an important proportion of genetic variability for BFT in the Italian Large White breed. In particular, two markers below the suggestive significance threshold were located both on *IGSF3* or close to *PKN2* on SSC4 and a few close blocks of SNPs with P<5.0E-05 (from about 65.1 - 65.4, 70.6 - 72.5, and 100.7 - 101.8 Mb) were located on SSC6 (Additional file 1: Table S1). As mentioned above, *FTO* is close to the marker at position 28215213 on SSC6. Single marker analysis using a few *FTO* SNPs in our previous large association study with BFT in Italian Large White pigs did not produce significant results [10]. However, subsequent haplotype analysis at this locus tended to confirm *FTO* as an important locus affecting fat deposition also in this pig breed [51].

## Conclusions

This study is the first genome wide association analysis for BFT in Italian heavy pigs. The targeted trait is of paramount importance for the Italian pig breeding industry that is devoted to the production of high quality dry-cured hams for which fat coverage is a key factor during the processing and curing steps [2,3]. The genetic dissection of BFT could open new perspectives to improve selection efficiency. In this study we applied a selective genotyping approach within the Italian Large White pig population to reduce the cost of genotyping without losing much power [26-31]. We took advantage of the large number of pigs that have been performance tested and genetically evaluated under the National selection program for this breed. The association analysis that compared SNP genotype frequencies between low BFT-EBV vs. high BFT-EBV groups identified 123 SNPs with P<5.0E-5 that were more densely represented in a few chromosomes known to harbor important QTLs for fat deposition traits. The quite large number of markers below this threshold (spread in different chromosome regions) might indirectly support the fact that many genes, each with a small-medium contribution, are involved in determining BFT, according to the classical definition of a quantitative trait.

Several significant or suggestively significant SNPs were close to genes whose function might be directly or indirectly related to energy metabolism and fat deposition. Many other cannot be easily linked to the targeted trait and might provide, if confirmed in following up studies, new evidence on this matter. Even if the annotation available in Pre-Ensembl for Sscrofa10.2 is preliminary, GO enrichment analysis indicated that neuronal genes might affect fat deposition in pig confirming and enlarging previous indications reported in humans [44].

Summarizing, as more information is becoming available in pigs on biological aspects of fat metabolism and deposition, it is more and more clear that this species could represent an attractive biomedical model for human obesity and associated diseases. Data here reported could give an insight over genetic mechanisms of fat metabolism and deposition that could be helpful in understanding also biology aspects of human obesity.

## Methods

### Animals and phenotypic traits

All animals used in this study were kept according to Italian and European legislation for pig production and all procedures described were in compliance with national and European Union regulations for animal care and slaughtering.

The national selection program of the Italian Large White breed is based on triplets of siblings from the same litter (two females and one castrated male) that are individually performance tested at the Central Test Station of the National Pig Breeder Association (ANAS) for the genetic evaluation of a boar from the same litter (sib-testing). Performance evaluation starts when the pigs are 30 to 45 days of age and it ends when the animals reach 155 ± 5 kg live weight. The nutritive level is *quasi ad libitum*, meaning that about 60% of the pigs are able to ingest the entire supplied ration. At the end of test, animals are transported to a commercial abattoir where they are slaughtered following standard procedures [52]. Then, backfat thickness is measured on the carcasses at the level of *Musculus gluteus medius*.

The association study was conducted following a selective genotyping approach (e.g. [26-31]). In this study we genotyped two extreme and divergent groups of Italian Large White gilts of these triplets (one female per triplet), performance tested in the period 1996–2007. Two-generation unrelated females (i.e. gilts with different and unique parents) were chosen according to their EBV for BFT (152 with most negative and 152 with most positive EBV) within a performance tested population of ~12000 pigs (details of EBV calculation are reported below). The two extreme groups were chosen ranking the animals according to their BFT EBV and then taking only the first unrelated gilts in the list (with the most positive or the most negative BFT EBV). BFT EBV used to choose the animals were recalculated for the whole performance tested population in 2007. Average BFT EBV in the negative and positive selected groups of pigs were −9.8 ± 1.6 mm and +6.6 ± 2.3 mm, respectively. Genotyped pigs were a subset of the 560 two-generation unrelated pigs used in our previous candidate gene association study [10].

### Genotyping

Genomic DNA was extracted from dried-blood by standard protocols. Based on quality control, all animals were used for genotyping using the PorcineSNP60 BeadChip [19] developed by Illumina according to manufacturer’s protocol [53].

### Data analyses

Estimated breeding values for BFT were calculated in the population using a BLUP-Multiple Trait-Animal Model that included the fixed effect of sex (considering the triplets of pigs from the same litter), batch on trial, inbreeding coefficient of the animal, interaction of sex by age at slaughtering, date of slaughtering and random effect of litter and animal. Three criteria were used to filter animals and SNP before association analysis: call rate >0.9 both at the 1) animal and 2) SNP level, and 3) MAF>0.05. Animals and SNPs that passed these filters were taken for association analysis treating the two groups as cases and controls. Full pedigree information available was used to obtain a kinship matrix. In order to correct for possible family-based stratification (see Additional file 2: Figure S1), the EIGENSTRAT method [54] was applied including the kinship matrix, and association tests were performed. All analyses were performed in R [55], using an option of the package GenABEL [56] for computing the test-statistics according to the EIGENSTRAT method, and the package kinship [57] for building the pedigree kinship matrix.

For *n* animals, the first *K*<*n* principal components, *c*_1_, …, *c*_*K*_, of the kinship matrix among the animals were used as axes of genetic variation. Let *g*_*ij*_ and *p*_*j*_ be the genotype at SNP *i* (*g*_*ij*_ = 0, 1 or 2) and the phenotype of animal *j*, respectively, a PC-based adjustment was performed on genotypes and phenotypes according to the following formulas:

(1) $$g_{\mathrm{ij}}^{*}=g_{\mathrm{ij}}-\beta_{1i}c_{1j}-..-\beta_{\mathrm{Ki}}c_{\mathrm{Kj}}$$

(2) $$p_{j}^{*}=p_{j}-\gamma_{1}c_{1j}-..-\gamma_{K}c_{\mathrm{Kj}}$$

where *c*_*kj*_ is the score of the *k*-th component on animal *j*, *β*_*ki*_ and *γ*_*k*_ are the partial regression coefficients for predicting the *i*-th genotype and the phenotype, respectively, on the basis of the *k*-th component (with *k* = 1, ., *K*).

The association test-statistic is computed as (*n* − *K* − 1)*r*_*i*_^2^, where

(3) $$r_{i}^{2}=\frac{{\left(\sum_{j=1}^{n}p_{j}^{*}g_{\mathrm{ij}}^{*}\right)}^{2}}{\sum_{j=1}^{n}{\left(p_{j}^{*}\right)}^{2}\sum_{j=1}^{n}{\left(g_{\mathrm{ij}}^{*}\right)}^{2}}$$

is the squared correlation coefficient between the *i*-th PC-adjusted genotype and PC-adjusted phenotype. As noted by Price et al. [54], this statistic is a generalization of the Armitage trend statistic for discrete genotypes and phenotypes.

Wellcome Trust Case Control Consortium significance thresholds, whose definition depends on the prior odds and power, were used in this study [30]. In addition, correction for multiple testing was achieved by using a False Discovery Rate approach [58]. For each chromosome, the expected proportion of SNPs with P<5.0E-5 was computed under the assumption of uniform distribution from the informative SNPs over the chromosome. This proportion was compared to the proportion of significant or suggestively significant markers actually observed on the same chromosome.

### Bioinformatics analyses

Mapping of the PorcineSNP60 BeadChip SNPs was obtained by using BWA [59] on the Sscrofa9.2 and Sscrofa10.2 genome assemblies as previously described [10] and confirmed using the BLAT analysis available at the Ensembl ( http://www.ensembl.org/Sus_scrofa/Info/Index) and Pre-Ensembl ( http://pre.ensembl.org/Sus_scrofa/Info/Index) databases (February 2012). Coordinates for the Sscrofa10 genome preliminary version (September 2010) were downloaded from the Animal Genome repository web site http://www.animalgenome.org/repository/. Identification of the closest genes to SNPs with P<5.0E-05 was obtained using Pre-Ensembl annotation of Sscrofa10.2 genome version and verified using Ensembl Sscrofa9.2 genome version (February 2012). Starting from the corresponding protein sequences retrieved from these databases, the corresponding gene symbols were extracted from NCBI Gene section ( http://www.ncbi.nlm.nih.gov/) and/or Uniprot ( http://www.uniprot.org/) databases (February 2012). Gene annotation was verified by BLAST analysis ( http://blast.ncbi.nlm.nih.gov/). Gene Ontology analysis was carried out using DAVID Bioinformatics Resources 6.7 ( http://david.abcc.ncifcrf.gov/[60]).

## Competing interests

The authors declare that they have no competing interests.

## Authors’ contributions

LF conceived and coordinated the study, analysed data and drafted the manuscript. GS, GG, DGC, and PLM performed statistical and bioinformatics analyses. ES carried out laboratory activities. LB, RC and VR coordinated and conceived the study. All authors reviewed and contributed to draft the manuscript. All authors read and approved the final manuscript.

## Supplementary Material

Additional file 1 **Table S1. **Suggestively significant SNPs (5.0E-07< P≤5.0E-05), their chromosome positions and their closest genes in Sscrofa10.2 (Pre-Ensembl). Notes are the same as those reported for Table 1. Click here for file

Additional file 2 **Figure S1. **Two-dimensional graphical representation of relatedness among animals based on a multidimensional scaling representation of pedigree-based kinship matrix. Different symbols are used to denote pigs with positive or negative backfat thickness estimated breeding values. Click here for file
