# Supplementary figures and images for: A genome wide association study for backfat thickness in Italian Large White pigs highlights new regions affecting fat deposition including neuronal genes
Source: BMC Genomics. 2012 Nov 15;13:583. doi: 10.1186/1471-2164-13-583 (PMC3499287; doi:10.1186/1471-2164-13-583)

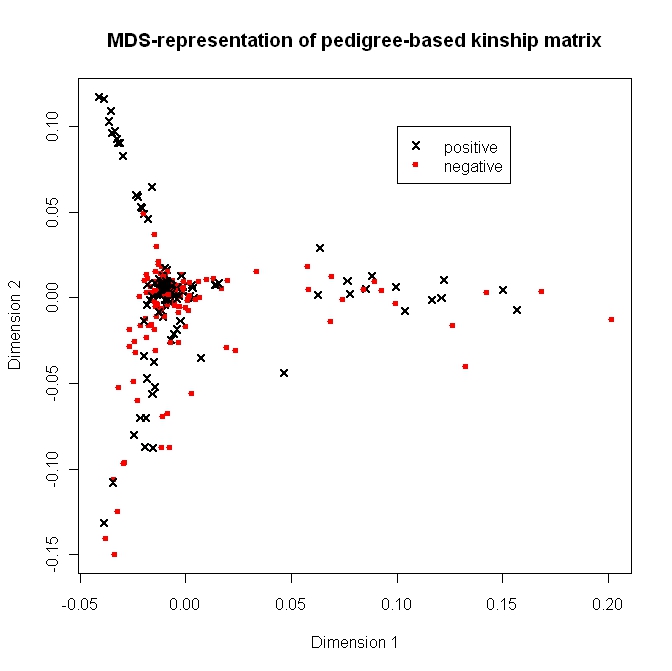

Supplement: Additional file 1 — Table S1. Suggestively significant SNPs (5.0E-07< P≤5.0E-05), their chromosome positions and their closest genes in Sscrofa10.2 (Pre-Ensembl). Notes are the same as those reported for Table 1. [file 1471-2164-13-583-S1.jpeg]
